# Clinical Outcomes of Modified Suture Buttons for Tibial Side Fixation in Anterior Cruciate Ligament Reconstruction: A Retrospective Comparative Study

**DOI:** 10.7759/cureus.64357

**Published:** 2024-07-11

**Authors:** Takuya Sakamoto, Manato Horii, Shotaro Watanabe, Ryu Ito, Ryuichiro Akagi, Hiroaki Hosokawa, Seiji Kimura, Satoshi Yamaguchi, Seiji Ohtori, Takahisa Sasho

**Affiliations:** 1 Department of Orthopedic Surgery, Graduate School of Medicine, Chiba University, Chiba, JPN; 2 Center for Preventive Medical Sciences, Chiba University, Chiba, JPN; 3 Department of Orthopedic Surgery, Graduate School of Medicine, Chiba University, Chiba, JPN, Chiba, JPN; 4 Department of Orthopedic Surgery, Oyumino Central Hospital, Chiba, JPN; 5 Department of Orthopedic Surgery, Toho University Sakura Medical Center, Chiba, JPN; 6 Graduate School of Medical and Pharmaceutical Sciences, Chiba University, Chiba, JPN; 7 Department of Orthopedic Surgery, Chiba University Graduate School of Global and Transdisciplinary Studies College of Liberal Arts and Sciences, Chiba, JPN; 8 Department of Orthopedic Surgery, Chiba University Hospital, Chiba, JPN

**Keywords:** arthroscopy, patient reported outcome measures, clinical outcomes, graft, tibial fixation, suture button, anterior knee laxity, anterior cruciate ligament

## Abstract

Introduction

Restoring knee joint stability and resuming sports activities are important objectives of anterior cruciate ligament (ACL) reconstruction. The maintenance of anterior knee stability after ACL reconstruction is contingent on graft tension. Various devices and techniques have been used to achieve robust tibial graft tendon fixation, and their advantages and disadvantages are established. However, a gold standard has not been established. Therefore, we aimed to determine whether anterior knee joint stability and clinical outcomes of graft tendon fixation could be improved using a recently modified suture button (MSB) compared with using an adjustable suspensory fixator (ASF) at 1 year after double-bundle ACL reconstruction.

Methods

This study retrospectively analyzed postoperative data derived from 79 patients at a single center between January 2016 and December 2021. The patients were assigned to groups that underwent double-bundle ACL reconstruction with tibial fixation using an MSB (n = 30) that maintains tension while tying sutures, or an ASF (n = 49). We then compared complications, clinical outcomes and knee joint stability at 1 year postoperatively. Rates of postoperative infection, graft rupture, implant removal and residual anterior knee laxity (AKL) were compared between the groups using chi-square tests. Patient-reported outcome measures (PROM) based on Forgot Joint Score-12, Knee Injury and Osteoarthritis Outcome, and Lysholm Knee scores were compared using Mann-Whitney U tests.

Results

One patient in the MSB group developed postoperative infection. Rates of graft rupture and implant removal in the MSB and ASF groups were 3.3% and 4.1%, and 3.6% and 10.2%, respectively. None of the PROMs differed between the groups. The proportions of postoperative AKL were 3.6% and 14.9% in the MSB and ASF groups, respectively. A trend towards lower postoperative AKL in the MSB group did not reach statistical significance (p = 0.25).

Conclusions

The incidence of AKL at a year after ACL reconstruction using the MSB was 3%. Postoperative AKL and clinical outcomes were comparable between the MSB and ASF groups. A low AKL rate and positive postoperative outcomes indicated that MSB could be an option for tibial-side fixation in ACL reconstruction.

## Introduction

Restoring knee joint stability and enabling a return to sport are critical objectives of anterior cruciate ligament (ACL) reconstruction. The maintenance of anterior knee stability after ACL reconstruction is contingent on the tension of the graft tendon complex and the position of the bony tunnel. The graft tendon complex should be postoperatively fixed at an appropriate initial tension that is not loose. Insufficient anterior knee joint stability adversely affects clinical performance, hampers return to sport, and predisposes the knee to cartilage damage, meniscal tears, and osteoarthritis [1,2]. Consequently, preventing graft loosening is a crucial determinant of successful ACL reconstruction.

Factors identified as causes of anterior knee joint laxity after ACL reconstruction include femoral tunnel implant loosening, graft substance elongation, and bony tunnel enlargement [3-6]. Furthermore, tibial fixation devices can contribute to graft loosening. Tibial fixation sites are characterized by low bone density and are particularly vulnerable to ACL reconstruction; thus, fixation must be robust [7,8]. Soft tissue grafts have been fixed with screw, adjustable suspensory fixator (ASF), double spike plate (DSP), and stapler during ACL reconstruction [9-11]. ASFs have been reported to loosen after graft tendon fixation in biomechanical studies [9].

A modified suture button (MSB) that can maintain tension while tying sutures has recently been introduced. The concept of the MSB secures an artificial ligament between outer and inner polyethylethylketone (PEEK) tubes, thus immobilizing graft ends close to the aperture of the bony tunnel. The MSB achieves stronger initial tension than DSP by allowing fixation with tension applied parallel to the tibial tunnel [12]. Nonetheless, anterior laxity or clinical outcomes associated with MSB after ACL reconstruction have not been reported.

This study aimed to determine whether knee joint stability and clinical outcomes 1 year after double-bundle ACL reconstruction were better when graft tendons were fixed using MSB than ASF. We speculated that MSB would outperform ASF in terms of anterior knee joint stability and clinical outcomes.

## Materials and methods

**Figure 1:**
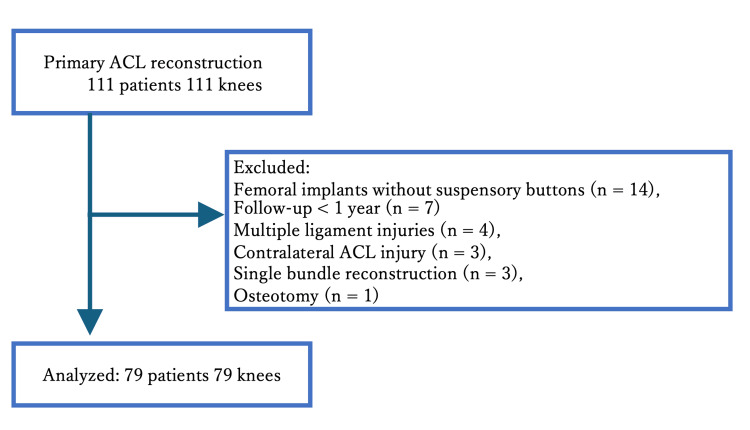
Flowchart of patients through the study ACL, anterior cruciate ligament

This retrospective observational study analyzed postoperative data from patients who underwent primary anatomical double-bundle ACL reconstruction at a single center between January 2016 and December 2021. The same techniques were applied during all ACL reconstructions using the semitendinosus tendon. The exclusion criteria comprised multiple ligament injuries, a history of ipsilateral or contralateral knee joint injury, osteoarthritis, rheumatoid arthritis, partial reconstruction, follow-up for < 1 year, osteotomy, and fixation devices other than ASF applied to the femoral side. Both the MSB and ASF groups underwent graft fixation on the tibial side using devices that were allocated based on timeframes (Figure 1).

**Figure 2:**
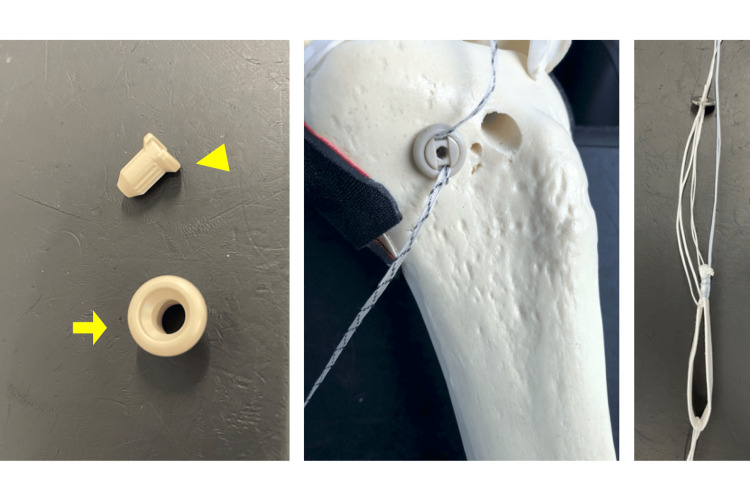
TensionLocTM System and TightRope®︎ ABS TensionLocTM System: The yellow triangle and arrow indicate inner and outer cylinders, respectively (left). TensionLocTM System: The outer cylinder is driven into the aperture of the tibial tunnel (center). TightRope®︎ ABS: An adjustable rope is applied inside the graft loop and a titanium button is attached (right). ABS, attachable button system TensionLoc System and TightRope ABS by Arthrex Corp., Naples, FL, USA.

We used a TightRope® Attachable Button System (ABS) (Arthrex Corp., Naples, FL, USA) that is an ASF, between 2016 and December 2018, then a TensionLocTM Tibial Fixation Implant (Arthrex Corp.) MSB from 2019 (Figure 2).

In a case with pain at the implant insertion site on the tibial side, the implant was removed.

The Ethics Committee at Chiba University approved this study (Approval ID: M10609) and waived the need for informed consent to analyze and publish historical innominate data. 

Surgical technique

Two knee surgeons (T.S. and R.A.) with > 10 years of experience, either as primary surgeons or as supervisors, conducted all knee operations. Anterolateral and anteromedial portals were created to gain visual access to the internal knee joint. The semitendinosus tendon was harvested after confirming a torn ACL and meniscal injury by incising the skin immediately above its tibial attachment and the sutured fascia. Muscle components were trimmed, and the semitendinosus tendon was divided into two sections with the attachment side on the anteromedial fibers and the muscle transition side on the posterolateral fibers. A bony tunnel was then created in the anatomical position. Initially, the femoral tunnel was established on the ACL footprint using an ACL femoral drill guide set at 100°, and a guide pin was inserted in the anteromedial tunnel position using the outside-in technique. The ACL femoral drill guide was set at 95° and a guide pin was inserted in the posterolateral tunnel position. After confirming the desired location, a bony tunnel was created using a retrograde drill that resulted in a 21-mm long socket with the same diameter as the graft. An ACL tibial drill guide was set at 60° for the tibial tunnel. An anteromedial tunnel was initially created using the anterior horn of the lateral meniscus and remaining ACL tissues as reference markers. The guide angle was changed to 55° to form a posterolateral tunnel. On the femoral side, the tendon was folded in half, and the loop side was prepared using a TightRope® II RT (Arthrex Corp.). Lateral tibial fixation proceeded using the TightRope® ABS or the TensionLocTM. The distal end of the TensionLocTM was prepared using five simple locking loop stitches with a 1.3-mm TapeLoop (Evolutis, Briennon, France) non-absorbable suture thread [13]. After passing the graft, joint tension was applied by flexing the knee from 0° to 90° 10 times. A tension meter was attached to both bundles and a 40 N tensile force was applied. If the pulling force was < 40 N, the sample was pulled again up to three times at 40 N [14]. The anteromedial bundle was then fixed at 20° of flexion, whereas the posterolateral bundle was secured in full extension, each with a traction force of 40 N. The adjustable rope in the TightRope® ABS was hooked and five simple locking loops were stitched, then closed. A titanium button with a 7-mm diameter was attached to the adjustable rope at the surface of the bony tunnel and secured while verifying that graft tension within the knee joint determined by arthroscopy was optimal for probing. Similar to the TensionLocTM, the knee joint was flexed and extended up to three times, and the tension of the graft tendon was reconfirmed arthroscopically by probing and readjusted with an adjustable loop if it had become loosened.

Rehabilitation protocol

A standardized rehabilitation protocol was implemented for all patients [15]. Isometric quadriceps training was initiated immediately after the surgical procedure. The load was initially limited to 30 kg for the first two weeks after surgery and gradually increased to full loading by three weeks postoperatively. Range of motion was restricted to 90 degrees of flexion until four weeks postoperatively. Brace was not used in all cases. Patients who could perform a standing up with both leg from a sitting position on a 10 cm high platform three months after surgery began jogging. Sport training was gradually reintroduced at seven months postoperatively for patients who could perform a standing up with affected single leg from a sitting position on a 20 cm high platform, with the aim of returning to sports competitions no earlier than 10 months postoperatively. Return to sports was permitted for patients who achieved at least 80% of the unaffected side’s performance in the single-leg hop test.

Data collection

Patient-related information (age, sex, injured side, body mass index (BMI), Tegner Activity Scale (TAS), and meniscus status), surgical duration and implant type, and postoperative complications (infection, graft rupture, and surgical implant removal) up to 1 year after surgery was obtained from medical records. To assess knee joint laxity, anterior tibial translation (ATT) was measured using a KS Measure (Nippon Sigmax Co., Tokyo, Japan) as described [16,17]. The orthopedic surgeons described above measured the values when maximum traction force was applied to the healthy and affected sides under anesthesia. Values that deviated by 3 mm on the same side were considered as errors and the measurements were repeated. The ATT was determined as the average of three measurements. Side-to-side differences in ATT were measured preoperatively and at 1 year postoperatively. Patients with a side-to-side difference in ATT > 3 mm were categorized as having anterior knee laxity (AKL) [18,19]. Patient-reported outcome measures (PROMs), such as the Forgot Joint Score-12 (FJS), Knee Injury and Osteoarthritis Outcome scores (KOOS), and Lysholm Knee Scores (LKS), were surveyed using questionnaires at 1 year postoperatively.

Statistical analysis

Summary statistics are shown as numbers (n) and proportions (%) for categorical data and means and standard deviations for continuous variables. Complication rates of infection, graft rupture, implant removal, and the proportions of patients with AKL were compared between the MSB and ASF groups using chi-squared tests. The FJS, KOOS, and LKS at 1 year postoperatively were compared using Mann-Whitney U tests because of non-normal distribution. Factors influencing the extent of AKL at 1 year after ACL reconstruction were assessed using multivariable logistic regression analysis. The presence or absence of AKL was an objective variable, and the explanatory variables comprised age, BMI, preoperative side-to-side difference in ATT, tibial side fixation implant (MSB or ASF), and the interval between injury and surgery. And multivariable linear regression analysis was performed using BMI, Tegner activity scale, preoperative waiting period, age, meniscus status, and tibial fixation implant type as explanatory variables and each PROM as objective variable. All data were statistically analyzed using EZR version 4.1.2 (R Foundation for Statistical Computing, Vienna, Austria). Values with p < 0.05 were considered statistically significant. Sample sizes were not statistically calculated.

## Results

During the study period, 111 patients underwent ACL reconstruction. We excluded 32 patients with multiple ligament injuries (n = 4), previous ACL injuries (n = 3), single-bundle reconstruction (n = 3), tibial osteotomy (n = 1), femoral implants other than ASF (n = 14), and follow-up < 1 year (n = 7). We finally analyzed data regarding 79 knees in 79 patients (Figure 1).

Participant demographics

Table 1 shows the patient characteristics. Age, sex, BMI, affected side, TAS, meniscus status, waiting period, preoperative side-to-side differences in ATT, and surgical duration did not significantly differ between the MSB (n = 30) and ASF (n = 49) groups. The follow-up was significantly shorter for the MSB than the ASF group (17.8 ± 6.9 vs. 34.6 ± 14.8, p < 0.01).

**Table 1:** Demographics of patients Data are shown as *numbers (n) with means ± standard deviations, †medians with ranges, and ‡numbers (n) and ratios (%). ASF, adjustable suspensory fixator; ATT, anterior tibial translation; BMI, body mass index; MSB, modified suture button

	MSB (n = 30)	ASF (n = 49)	p
Age (years)*	29.0 ± 12.8	27.9 ± 11.4	0.67
Sex, n (%)			0.16
Male	15 (50.0)	16 (32.7)	
Female	15 (50.0)	33 (67.3)	
BMI (kg/m^2^)*	23.0 ± 3.7	23.8 ± 6.2	0.68
Tegner Activity Scale*	5.8 ± 2.1	6.0 ± 1.9	0.69
Wait period (days)^†^	43 (14-467)	93 (13-3906)	0.09
Meniscus injury, n (%)^‡^			0.37
Tear	21 (70.0)	32 (65.3)	
Intact	9 (30.0)	17 (34.7)	
Preoperative ATT (mm)*	4.3 ± 3.5	3.4 ± 3.0	0.24
Operation time (min)*	153.4 ± 49.1	151.8 ± 39.1	0.87
Follow-up (months)*	17.8 ± 6.9	34.6 ± 14.8	<0.01

Complications

One patient in the MSB group experienced a postoperative infection within the first year. The graft rupture rates within 1 year were 3.3% and 4.1% in the MSB and ASF groups, respectively (Table 2). The removal rates were 3.3% and 10.2% at 1 year after surgery in the MSB and ASF groups, respectively, but the difference did not reach statistical significance (p = 0.40).

**Table 2:** Complication rates at 1 year postoperatively ASF, adjustable suspensory fixator; MSB, modified suture button

	MSB (%) (n = 30)	ASF (%) (n = 49)	p
Infection (%)	3.3	0.0	0.38
Graft rapture (%)	3.3	4.1	1.00
Implant Removal (%)	3.3	10.2	0.87

Patient-reported outcome measures (PROM) 

Table 3 presents the clinical outcomes of the patients. The FJS, KOOS, and LKS did not significantly differ between the groups.

**Table 3:** PROM and AKL rate at 1 year postoperatively FJS-12 (n = 66)/ KOOS (n = 73)/ LKS n = 72)/AKL (n = 74) Data are shown as means ± standard deviations and prevalence ADL, activities of daily living; AKL, anterior knee laxity; ASF, adjustable suspensory fixator; FJS, forgotten joint score-12; KOOS, knee injury and osteoarthritis outcome score; LKS, Lysholm knee scoring scale; MSB, modified suture button; QOL, quality of life

	MSB (n=28)	ASF (n=47)	p
FJS	84.7 ± 22.4	83.1 ± 19.8	0.39
KOOS			
Pain	92.1 ± 10.7	91.2 ± 10.2	0.53
Symptoms	88.3 ± 12.5	87.0 ± 12.8	0.95
ADL	97.2 ± 6.2	96.3 ± 6.1	0.42
Sport	83.9 ± 21.4	81.0 ± 18.0	0.30
QOL	75.9 ± 26.9	77.2 ± 19.3	0.64
LKS	90.2 ± 13.8	91.5 ± 8.6	0.82
AKL (%)	3.6	14.9	0.24

Factors influencing postoperative PROM

Multivariable linear regression analysis for each PROM showed that the type of tibial implant had no significant effect (no table).

Anterior knee laxity (AKL)

The ratios of patients with postoperative AKL were 3.6% and 14.9% in the MSB and ASF groups, respectively, but the difference was not statistically significant (p = 0.25; Table 3).

Factors influencing postoperative AKL

Multivarable Logistic regression analysis of postoperative AKL did not reveal any independent risk factors (Table 4).

**Table 4:** Multivariable logistic regression analysis to predict AKL at 1 year after ACL reconstruction AKL: anterior knee laxity, BMI: body mass index, MSB: modified suture button, CI: Confidence Interval, ATT: anterior tibial translation, ACL: anterior cruciate ligament

	Odds ratio	95% CI	p-value
Intercept	0.01	0.00 - 0.18	0.01
Age	0.97	0.89 - 1.06	0.51
BMI	1.16	0.98 - 1.37	0.08
Preoperative ATT	1.38	0.99 – 1.91	0.06
Tibial side implant (MSB)	8.10	0.68 - 96.80	0.10
The period from injury to surgery	0.99	0.98-1.00	0.18

## Discussion

The key finding of this study was that ACL reconstruction using an MSB that maintained tension while tying sutures yielded similar postoperative anterior tibial laxity and PROMs to those of ASF. This study is the first to investigate the clinical outcomes of ACL reconstruction using MSBs. The main features of the MSB are that it is made of PEEK, fixation is achieved using clamps between the outer and inner tubes at bone tunnel openings, and surgeons can accurately set fixation tension. PEEK has been applied as an interference screw in ACL reconstruction for graft fixation [20]. Its advantages include low susceptibility to artifacts in images and minimal risk of sinking into the bone because it has a similar stiffness to that of the natural bone cortex [21-23]. The MSB achieves fixation closer to the aperture by driving part of the outer tube into the bone and inserting an artificial ligament between the outer and inner tubes. Fixation close to the aperture reduces windshield wiper and bungee effects [12].

The tibial fixture type and AKL development in patients who underwent ACL reconstruction are associated. The tension of the ASF tended to be lower than that of an interference screw and ASF in a biomechanical study of bovine tibia [24]. A biomechanical study of suspensory fixation showed that the adjustable mechanism loosens under repeated tensile stress [9]. The outcomes of tibial-side graft fixtures and knee laxity are comparable between interference screws and ASF when using allografts [25,26]. A stapler as an adjunct to the interference screw provides stronger fixation but carries the risk of future removal [27]. The MSB was stiffer in repeated test cycles and had more linear stiffness to failure than DSP tibial fixation in cadavers with bone-tendon-bone grafts [28]. The AKL of joints at 1 year after ACL reconstruction differed by ≥ 3 mm in 8%-46% of patients [20,29]. The present study found no significant differences in AKL between MSB and ASF fixation at 1 year postoperatively. The AKL rate at 1 year after ACL reconstruction was 3.6%, which is consistent with previous findings.

The relationship between the tibial fixture type and postoperative clinical outcomes has been investigated. The PROMs in the present study were good, with KOOS scores for symptoms (88.3), pain (92.1), activities of daily living (ADLs) (97.2), sport (83.9), quality of life (QOL) (75.9) and LKS (90.2) at 1 year after ACL reconstruction in the MSB group. We believe that MSB could be an option for ACL reconstruction because of the low incidence of AKL and favorable clinical outcomes.

The proportion of patients requiring hardware removal in the MSB group was 3.3%, which was similar to previous findings [30]. All implants were removed from patients with tibial implant irritation. Prospective studies of tibial fixation devices have found that 11% of patients require staple fixation removal due to irritation at the tibial fixation site, whereas such irritation did not occur when absorbable screws were used at the tibial fixation site [1,30]. We assumed that MSB has fewer postoperative irritation symptoms owing to less protrusion into the bone surface.

The retrospective design, short-term follow-up, and insufficient sample size (power analysis indicated a need for 115 patients per group) are limitations of the present study. Not matching the meniscus status and activity level between the two groups is also a limitation, but there was no statistical difference in patient demographics. However, we investigated knee joint stability and are the first to describe the clinical outcomes and AKL after ACL reconstruction using an MSB. Further studies with larger samples and longer follow-ups are required to validate our findings.

## Conclusions

This study was the first to investigate the clinical outcomes of ACL reconstruction using MSB. The main features of the MSB were that fixation is achieved using clamps between the outer and inner tubes at bone tunnel openings, and surgeons can accurately set fixation tension.

The rate of AKL at 1 year after ACL reconstruction using an MSB was low at 3.6%, which was not inferior to ASF. PROMs at 1 year postoperatively were also comparable to ASF. The tibial fixation of the grafted tendon is one of the critical parts of the ACL reconstruction, and these results suggested an MSB it could be an option.

## References

[1] Ahn JH, Kim JG, Wang JH, Jung CH, Lim HC (2012). Long-term results of anterior cruciate ligament reconstruction using bone-patellar tendon-bone: an analysis of the factors affecting the development of osteoarthritis. Arthroscopy.

[2] Shelbourne KD, Benner RW, Gray T (2017). Results of anterior cruciate ligament reconstruction with patellar tendon autografts: objective factors associated with the development of osteoarthritis at 20 to 33 years after surgery. Am J Sports Med.

[3] Chiang ER, Chen KH, Chih-Chang Lin A (2019). Comparison of tunnel enlargement and clinical outcome between bioabsorbable interference screws and cortical button-post fixation in arthroscopic double-bundle anterior cruciate ligament reconstruction: a prospective, randomized study with a minimum follow-up of 2 years. Arthroscopy.

[4] Lind M, Nielsen T, Sørensen OG, Mygind-Klavsen B, Faunø P, Leake-Gardner S (2020). Bone ingrowth into open architecture PEEK interference screw after ACL reconstruction. J Exp Orthop.

[5] Mayr R, Smekal V, Koidl C (2020). ACL reconstruction with adjustable-length loop cortical button fixation results in less tibial tunnel widening compared with interference screw fixation. Knee Surg Sports Traumatol Arthrosc.

[6] Noyes FR, Huser LE, Ashman B, Palmer M (2019). Anterior cruciate ligament graft conditioning required to prevent an abnormal Lachman and pivot shift after ACL reconstruction: a robotic study of 3 ACL graft constructs. Am J Sports Med.

[7] Bartz RL, Mossoni K, Tyber J, Tokish J, Gall K, Siparsky PN (2007). A biomechanical comparison of initial fixation strength of 3 different methods of anterior cruciate ligament soft tissue graft tibial fixation: resistance to monotonic and cyclic loading. Am J Sports Med.

[8] Scheffler SU, Südkamp NP, Göckenjan A, Hoffmann RF, Weiler A (2002). Biomechanical comparison of hamstring and patellar tendon graft anterior cruciate ligament reconstruction techniques: the impact of fixation level and fixation method under cyclic loading. Arthroscopy.

[9] Barrow AE, Pilia M, Guda T, Kadrmas WR, Burns TC (2014). Femoral suspension devices for anterior cruciate ligament reconstruction: do adjustable loops lengthen?. Am J Sports Med.

[10] Petre BM, Smith SD, Jansson KS, de Meijer PP, Hackett TR, LaPrade RF, Wijdicks CA (2013). Femoral cortical suspension devices for soft tissue anterior cruciate ligament reconstruction: a comparative biomechanical study. Am J Sports Med.

[11] Sonnery-Cottet B, Rezende FC, Martins Neto A, Fayard JM, Thaunat M, Kader DF (2014). Arthroscopically confirmed femoral button deployment. Arthrosc Tech.

[12] Kimura M, Nakase J, Asai K, Yoshimizu R, Kanayama T, Tsuchiya H (2022). Tibial graft fixation methods and bone tunnel enlargement: a comparison between the TensionLoc implant system and the double-spike plate. Asia Pac J Sports Med Arthrosc Rehabil Technol.

[13] Sasho T, Sasaki T, Hoshi H (2018). Evaluating different closed loop graft preparation technique for tibial suspensory fixation in ACL reconstruction using TightRope™. Asia Pac J Sports Med Arthrosc Rehabil Technol.

[14] Jiang D, Ao YF, Jiao C (2019). The effect of cyclic knee motion on the elongation of four-strand hamstring autograft in anterior cruciate ligament reconstruction: an in-situ pilot study. BMC Musculoskelet Disord.

[15] Ono Y, Sato Y, Mukai H (2021). Randomized comparative study of suspension femoral fixation device in graft position maintenance in anterior cruciate ligament reconstruction: EndoButton CL vs TightRope RT. Asia Pac J Sports Med Arthrosc Rehabil Technol.

[16] Boutsiadis A, Panisset JC, Devitt BM, Mauris F, Barthelemy R, Barth J (2018). Anterior laxity at 2 years after anterior cruciate ligament reconstruction is comparable when using adjustable-loop suspensory fixation and interference screw fixation. Am J Sports Med.

[17] Kocher MS, Steadman JR, Briggs KK, Sterett WI, Hawkins RJ (2004). Relationships between objective assessment of ligament stability and subjective assessment of symptoms and function after anterior cruciate ligament reconstruction. Am J Sports Med.

[18] Asagumo H, Kimura M, Kobayashi Y, Taki M, Takagishi K (2007). Anatomic reconstruction of the anterior cruciate ligament using double-bundle hamstring tendons: surgical techniques, clinical outcomes, and complications. Arthroscopy.

[19] Fu FH, Shen W, Starman JS, Okeke N, Irrgang JJ (2008). Primary anatomic double-bundle anterior cruciate ligament reconstruction: a preliminary 2-year prospective study. Am J Sports Med.

[20] Bressy G, Brun V, Ferrier A (2016). Lack of stability at more than 12 months of follow-up after anterior cruciate ligament reconstruction using all-inside quadruple-stranded semitendinosus graft with adjustable cortical button fixation in both femoral and tibial sides. Orthop Traumatol Surg Res.

[21] Fang CH, Li M, Zhang YF, Liu H (2021). Extra-articular migration of PEEK interference screw after anterior cruciate ligament reconstruction: a report of two cases. BMC Musculoskelet Disord.

[22] Seaman S, Kerezoudis P, Bydon M, Torner JC, Hitchon PW (2017). Titanium vs. polyetheretherketone (PEEK) interbody fusion: Meta-analysis and review of the literature. J Clin Neurosci.

[23] Shumborski S, Heath E, Salmon LJ, Roe JP, Linklater JP, Facek M, Pinczewski LA (2019). A randomized controlled trial of PEEK versus titanium interference screws for anterior cruciate ligament reconstruction with 2-year follow-up. Am J Sports Med.

[24] Mayr R, Heinrichs CH, Eichinger M, Coppola C, Schmoelz W, Attal R (2015). Biomechanical comparison of 2 anterior cruciate ligament graft preparation techniques for tibial fixation: adjustable-length loop cortical button or interference screw. Am J Sports Med.

[25] Colombet P, Graveleau N, Jambou S (2016). Incorporation of hamstring grafts within the tibial tunnel after anterior cruciate ligament reconstruction: magnetic resonance imaging of suspensory fixation versus interference screws. Am J Sports Med.

[26] Lubowitz JH, Schwartzberg R, Smith P (2015). Cortical suspensory button versus aperture interference screw fixation for knee anterior cruciate ligament soft-tissue allograft: a prospective, randomized controlled trial. Arthroscopy.

[27] Hill PF, Russell VJ, Salmon LJ, Pinczewski LA (2005). The influence of supplementary tibial fixation on laxity measurements after anterior cruciate ligament reconstruction with hamstring tendons in female patients. Am J Sports Med.

[28] Jernick M, Borden PS, Seager A, McGarry MH, Adamson GJ, Lee TQ (2023). Biomechanical evaluation of TensionLoc versus the double spike plate for ACL graft tibial fixation. Orthop J Sports Med.

[29] Mohtadi N, Chan D, Barber R, Oddone Paolucci E (2015). A randomized clinical trial comparing patellar tendon, hamstring tendon, and double-bundle ACL reconstructions: patient-reported and clinical outcomes at a minimal 2-year follow-up. Clin J Sport Med.

[30] Freedman KB, D'Amato MJ, Nedeff DD, Kaz A, Bach BR Jr (2003). Arthroscopic anterior cruciate ligament reconstruction: a metaanalysis comparing patellar tendon and hamstring tendon autografts. Am J Sports Med.

